# Editorial: Artificial intelligence and imaging for oncology

**DOI:** 10.3389/fonc.2025.1547968

**Published:** 2025-02-06

**Authors:** Yuxiang Zhou, Zhimin Li, Poonam Yadav

**Affiliations:** ^1^ Department of Radiology, Mayo Clinic at Arizona, Phoenix, AZ, United States; ^2^ Department of Radiology, Feinberg School of Medicine, Northwestern University, Chicago, IL, United States; ^3^ Department of Radiation Oncology, Feinberg School of Medicine, Northwestern University, Chicago, IL, United States

**Keywords:** artificial intelligence, CT, MRI, ultrasound, PET, pathological image, deep learning, radiomics

## Introduction

Recent advancements in artificial intelligence (AI) and imaging technologies have significantly transformed the diagnostic and therapeutic landscapes of oncology (1–3). Cutting-edge imaging modalities, such as CT, PET, US, and MRI, are being increasingly utilized for tumor imaging (4–7), with emerging interdisciplinary fields like MR-LINAC gaining considerable traction (8, 9). This accelerating convergence of imaging and therapy in oncology highlights the urgent need to further explore the role of AI and imaging across various oncology specialties, including radiation therapy, to enhance cancer care. In response to this need, the topic titled “Artificial Intelligence and Imaging in Oncology” has been proposed, bringing together 19 contributions from 149 authors/experts in the field. These contributions delve into the potential of AI and imaging in tumor diagnosis and treatment, explore emerging AI-driven models for oncology diagnosis and prediction, and highlight the extraction of quantitative features from medical images to predict tumor behavior, therapy response, and patient prognosis.

## AI and imaging in tumor diagnosis and treatment

AI is revolutionizing cancer diagnosis and treatment by enhancing the accuracy and efficiency of medical image analysis. By analyzing medical images like CT scans, MRIs, and X-rays, AI algorithms can detect tumors earlier, differentiate between benign and malignant growths, and assist in treatment planning and monitoring.

Shao et al. demonstrated the potential of radiomics-based nomograms in enhancing the diagnostic capabilities of CT imaging. By extracting quantitative features from CT images, these nomograms can more accurately differentiate between conditions like intravenous leiomyomatosis and uterine leiomyoma, offering a significant clinical advantage over traditional CT image interpretation.

Zeng et al. explored the potential of fusing multimodal imaging with ultrasound to enhance the accuracy of interventional diagnostic procedures. By integrating machine learning techniques, they demonstrated the clinical utility of this approach in guiding percutaneous biopsies of liver and adjacent organs, leading to improved diagnostic success rates.

Yu et al. showed that UNet based deep learning models when applied to positional CT and CBCT images and extracted radiomics features show clinical significance of CBCT images. The work showed that dice coefficient results of CBCT are within 85% of the results of pCT for rectal cancer imaging. CBCT images are frequently utilized on radiation treatment modalities.

Yang et al. explored the potential of combining ultrasound imaging with radiomics analysis to differentiate small clear cell renal cell carcinoma (ccRCC) from renal angiomyolipoma (RAML). By developing and validating models that incorporate both clinical and radiomic features, the study seeks to enhance diagnostic accuracy and support more precise treatment decisions for patients with small renal tumors. The findings suggest that this innovative approach could significantly improve the clinical utility of ultrasound in managing renal neoplasms.

Wen et al. explored an innovative approach to differentiate benign from malignant head and neck tumors using synthetic MRI in conjunction with FSE-PROPELLER DWI. In their study, the authors employed both synthetic MRI and FSE-PROPELLER diffusion-weighted imaging (DWI) to investigate the characteristics of malignant and benign head and neck tumors. The study involved 48 subjects, who were retrospectively classified into malignant and benign groups. The results were promising, demonstrating that both synthetic MRI and FSE-PROPELLER DWI can quantitatively distinguish malignant from benign tumors based on T2 and ADC values. Notably, combining T2 and ADC values provided improved accuracy in tumor differentiation.

Liu et al. focused on the differential diagnosis of two common adrenal tumors that are often misdiagnosed in clinical practice. Their research utilized radiomics techniques, enhancing diagnostic accuracy without the need for enhanced CT scans.

Haghshomar et al. reviewed recent advancements in the application of artificial intelligence (AI) in liver oncology imaging. They specifically highlighted the evolution of manual radiomic techniques and the increasing use of deep learning-based representations for more accurate assessments. They demonstrated radiomics, a framework that complements conventional radiological interpretation, has emerged as a powerful tool for extracting and quantifying texture characteristics derived from tumor heterogeneity.

## Emerging AI-driven models for oncology diagnosis and prediction

Emerging AI-driven models are revolutionizing oncology by enabling earlier and more accurate cancer diagnosis. By analyzing vast amounts of medical data, these models can identify subtle patterns and predict disease progression, leading to more personalized and effective treatment plans.

Xie et. al., conducted a study to establish this deep learning (DL) driven Artificial intelligence (AI) system for predicting malignant STTs based on US images and clinical indexes of the patients. The AI system could extract more morphological features of the system and heatmaps of images for classifying malignant soft tissue tumors. The system utilized a ResNet based architect on both grey scale and color ultrasound images for tumor feature extraction. The model can assist clinicians in diagnosing soft tissue tumors.

Ullah et al. studied brain tumor on MRI images diligently incorporating linear stretching in contrast enhancement and data augmented images fed to variants of efficient Net and Inception ResNet. The study utilized bayesian optimization on their deep learning process and showed an accuracy improvement over limited clinical dataset for brain tumor classification. The study showed that cubic SVM can increase accuracy by 0.5% over a bilayered neural network.

Wang et al. studied performance of MAMIL Net by histologic features in predicting breast cancer in sentinel lymph node, differentiating lung adenocarcinoma from squamous cell carcinoma, and predicting therapeutic response of high-grade ovarian serous carcinoma by retrospective case series. They found that MAMILNet performed excellent for lung cancer, good for breast cancer and fair for ovarian cancer based on AUC and accuracy values, suggesting that this learning framework has the potential in disease diagnosis and prognosis.

**Figure 1:**
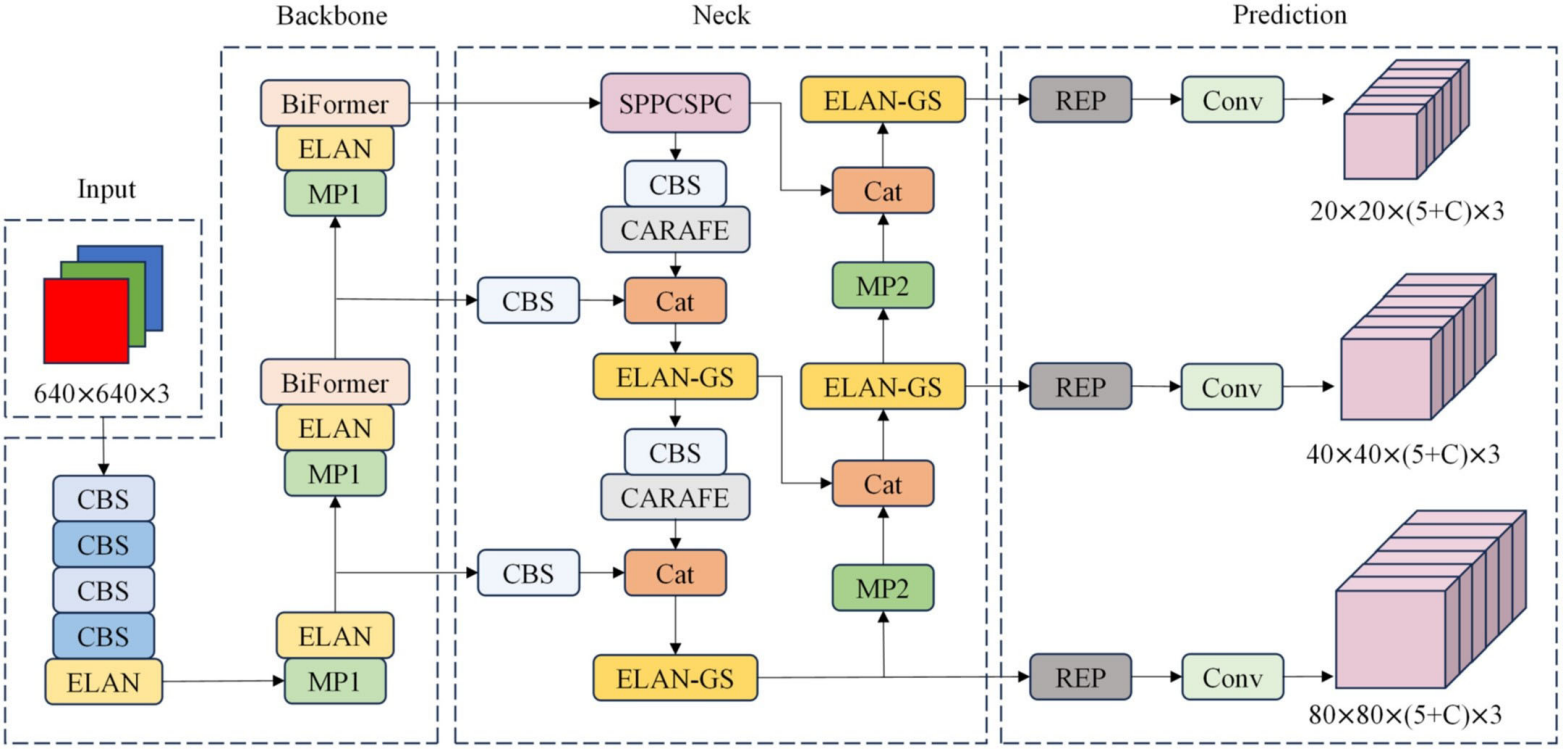
PI-YOLO Network architecture, including Input, Backbone, Neck, and Prediction. C in the Prediction module is the number of categories in the dataset.

Li et al. developed PI-YOLO, a novel deep learning model designed for automated blood vessel detection in pathology images. This model effectively addresses the challenges posed by complex backgrounds, small targets, and dense distributions in these images. By incorporating the BiFormer attention mechanism, PI-YOLO efficiently captures long-range dependencies and reduces computational costs. Additionally, the use of GSConv convolution further enhances the model’s performance by reducing parameters and improving inference speed. The results demonstrate that PI-YOLO achieves a significant mAP of 87.48%, outperforming existing methods. This advancement in automated blood vessel detection holds significant medical value, particularly in the field of anti-tumor vascular therapy research. **Figure 1** showed a typical network framework including four main components: Input, Backbone, Neck, and Prediction.

Krishnapriya and Karuna performed a study to show that deep learning-based YOLO architecture can predict bounding boxes for prediction and have added enhancements compared to its peers for analogous inference tasks. The grab cut algorithm assisted segmentation is likely to improve dice coefficients by 0.1 in the presented dataset and is worth exploring in brain tumor detection pipelines.

Awais et al. presented a novel decision support system for identifying acute lymphoblastic leukemia (ALL). By combining techniques like neighborhood pixel transformation, transfer learning from deep neural networks, and a customized binary Grey Wolf Algorithm for feature optimization, the system achieves outstanding accuracy in both binary and subtype classification of ALL. This approach holds great promise in aiding medical professionals in the early and precise diagnosis of this aggressive leukemia, leading to better patient outcomes.

Houssein et al. investigated a new and efficient deep learning technique for classifying white blood cells (WBCs) in blood smear images, crucial for diagnosing leukemia. Leveraging DenseNet-161 and optimizing the training process with cyclical learning rates, the method achieves exceptional accuracy in classifying various WBC types, surpassing current state-of-the-art techniques. This innovative approach has great potential to aid medical professionals in the early and accurate diagnosis of leukemia, ultimately improving patient outcomes.

## Radiomics and radiogenomics for predicting tumor behavior, treatment response, and patient outcomes

Radiomics and radiogenomics are emerging fields that extract quantitative features from medical images to predict tumor behavior, treatment response, and patient outcomes. By analyzing these features, clinicians can make more informed decisions about treatment strategies and monitor disease progression.

Lan et. al., Radiomics has shown promising applicability in cancer prediction, especially in recurrence. Lan et al. utilized ROIs delineated on CT images for extracting over 1100 radiomic features. To incorporate post-surgical data they used ten features based on relevance. This work shows employing clinical data over imaging parameters can be effectively used for predicting stage 1 lung adenocarcinoma prediction.

Mao et al. explored a novel radiomic nomogram that effectively differentiates parotid pleomorphic adenoma (PA) from adenolymphoma (AL) using grayscale ultrasonography. By combining advanced image analysis techniques with machine learning algorithms, this non-invasive nomogram provides a highly accurate method for distinguishing between these two common parotid gland tumors. This innovative approach has the potential to greatly enhance diagnostic precision and guide more effective treatment planning for patients with parotid gland lesions.

Liu et al., demonstrated the potential of radiomics-based machine learning models using 18F-FDG PET/CT imaging data to distinguish between adenocarcinoma and squamous cell carcinoma in cervical cancer. By extracting and analyzing numerous quantitative features from medical images, these models offer valuable insights into tumor biology and assist in personalized treatment planning. The study highlights the promising role of radiomics in improving the diagnosis and management of cervical cancer.

Hu et al. introduced an innovative approach for predicting microvascular invasion (MVI) in hepatocellular carcinoma (HCC), a critical factor influencing the disease’s aggressiveness. By integrating MRI imaging data with microRNA analysis, the researchers developed a radiogenomics nomogram that significantly outperforms existing models. This tool offers a promising path for more accurate risk assessment and personalized treatment strategies for HCC patients. With its high sensitivity and specificity, the nomogram shows great potential in improving clinical decision-making and enhancing patient outcomes.

Hu et al., explored a novel approach to testicular tumor diagnosis using computed tomography (CT) texture analysis (CTTA). This technique involves analyzing the texture patterns within CT images to identify subtle differences between benign and malignant tumors. By extracting specific texture features, researchers were able to develop machine learning models that can accurately classify tumors with high precision. One of the most promising findings of this study is the ability of CTTA to differentiate between primary testicular lymphoma and other malignant tumors. This distinction is particularly important as it can influence treatment strategies. Additionally, CTTA can help identify seminoma, the most common type of testicular germ cell tumor, from other types of germ cell tumors.

## Summary

This Research Topic explores the transformative role of artificial intelligence (AI) and imaging advancements in oncology, focusing on how these technologies are reshaping the field. The articles highlight the growing integration of AI and imaging across various oncology specialties, demonstrating their potential to revolutionize cancer diagnosis, treatment planning, and prognostication. By leveraging cutting-edge imaging modalities, such as CT, PET, US, and MRI, along with AI-driven models, these innovations are improving the accuracy of tumor detection, enabling personalized treatment strategies, and predicting patient outcomes with greater precision. The Research Topic emphasizes the need for continued research and development in these areas, with the promise of enhancing patient care and outcomes across diverse cancer types.
